# Cross-Sectional Survey of Healthcare Provisions for Female Tuberculosis Patients in Specialized Pulmonary Division from Low Socioeconomic Class in Lahore, Pakistan

**DOI:** 10.7759/cureus.1133

**Published:** 2017-04-03

**Authors:** Ahsan Zil-E-Ali, Muhammad Aadil, Syed A Abbas, Anas Ahmad, Sami Ur Rehman, Fatima Zil-E-Ali, Humera Karim, Saadia Shafi, Ammara I Khan

**Affiliations:** 1 Surgery, Pakistan Institute of Medical Sciences, Islamabad, Pakistan; 2 Department of Psychiatry and Mental Health, Rush University Medical Center; 3 Department of Internal Medicine, Fatima Memorial Hospital; 4 Department of General Medicine, Avicenna Hospital, Lahore.; 5 Medical Student 3, Lahore Medical and Dental College; 6 Department of Psychiatry, Shifa College of Medicine

**Keywords:** tuberculosis, infectious diseases, socioeconomic status

## Abstract

**Objectives:**

This study aimed to investigate various healthcare provisions for women affected with tuberculosis (TB) from low socioeconomic status and their health seeking behaviors, also whether or not patients feel stigmatized about their disease.

**Introduction:**

Pulmonary tuberculosis has more prevalence in Pakistan as compared to western countries where it occurs predominantly in the immunocompromised individuals and immigrants of certain countries. It is a contagious disease and Pakistan stands at the fifth position with maximum reported cases each year.

**Methods:**

A cross-sectional study was carried at Gulab Devi Hospital, a public sector hospital located in Lahore, through a questionnaire-based survey followed by interviewing all participants. Two hundred seventy-seven female patients, who were already diagnosed with pulmonary tuberculosis, were included in the study. The sample was drawn by non-probability, convenience sampling. Literacy, a major contributor to socioeconomic status, was taken primary criteria to select the sample for the study.

**Results:**

The study shows that literacy of patients has no impact on whether they feel stigmatized due to their disease as 42% (45 out of 108) of the literate women felt stigmatized while 39% (65 out of 169) of illiterate women also presented with similar feelings. Furthermore, the research also showed that these patients have no effect on requiring permission for going to the health facility as the study revealed that 62% (67 out of 108) in the literate women required permission while 67% (113 out of 169) illiterate women required permission.

**Conclusion:**

Pakistani population must be educated about TB and factors associated with the progression and consequences of the disease. It was noted that even educated people feel embarrassed when they develop symptoms of TB, thereby causing the unprecedented delay in effective disease management. To conclude, TB clinic should be opened in each community so that people have easy access to treatment of the disease.

## Introduction

Tuberculosis (TB) caused by the Mycobacterium tuberculosis is a highly contagious infection. The majority of the cases are of pulmonary tuberculosis but it can also involve other systems of the body. It is an airborne communicable disease transmitted through inhalation of infected droplets from the air [1].

A worldwide survey done in the year 2009 regarding the statistical data of tuberculosis stated that globally the prevalence rate of TB is 1.4 billion. The incidence rate for this disease has been estimated to be 9.4 million. Sixty-four percent of the diagnosed tuberculosis patients were males. However, the male to female ratio for the prevalence of TB differed among the different age groups and no significant difference between the sexes has been noted in the children cases of TB. However, more females have been diagnosed with TB amongst the young adults. On the other hand, in the population aged forty and above, males are more likely to be infected with TB [2].

TB is a worldwide endemic, though the highest rates per capita are in Africa, a quarter of all TB cases, half of all incident cases are in six Asian countries including China, India, Indonesia, Pakistan, and the Philippines. Many factors together affect the prevalence of multiple TB cases diagnosed all around the world every year. In developed countries, such as America, United Kingdom and immigrants from other developing countries, TB is more common in the elderly age group which can be easily associated with decreased immunity in the same age group. For instance, only 14,000 cases of TB were recorded in the year 2009. However, in developing countries, such as Pakistan and India, this disease is found to be more common in infants and adults affecting nearly sixty-seven hundred thousand people in Pakistan [3].

Pakistan has been ranked fifth by the World Health Organization (WHO) in terms of the number of TB cases being reported [3]. TB affects men more than it affects the women in Pakistan, which is also consistent with the worldwide distribution of TB cases. Studies conducted on the provincial level in Pakistan revealed 72,000 TB cases in Sindh, 115,000 in Punjab, 26,000 cases in Baluchistan, and 40,000 cases in Khyber-Pakhtunkhwa. From the last five years, progress has been made in the detection of TB cases from 13% in 2002 to 67% in 2007, and it has increased to 70%, the target proposed by the WHO [4-5]. A rise in diagnosing TB cases has been largely observed due to increase in the involvement of private sectors and community volunteering, although much work still needs to be done in distant and non-urban populations. Pakistan has developed National Tuberculosis Control Program (NTCP) in accordance with the WHO guidelines which aims to control TB by reducing the human reservoir by early case detection, improving the immunity of people by mass vaccinations, and reduction in the transmission of the causative agent by adequate public education [6-7].

## Materials and methods

A cross-sectional study was carried in the city of Lahore at the Division of Pulmonology at Gulab Devi Chest Hospital, a public sector healthcare facility during December 2016 to February 2017. A questionnaire was filled by an interview taken in Urdu and Punjabi language, according to the understanding of the patients.

The sample size was drawn by non-probability, convenience sampling from the consecutive reported patients. Two hundred seventy-seven female indoor department, pulmonary tuberculosis patients were included in the study, all patients ranging in age from 14 to 80 years old, residents of non-urban areas with a monthly home income less than Rs.10,000 (approximately US$ 100). The selected sample was further classified on the literacy level which was 'minimum of high school pass' of the patients. The sample was irrespective of their marital status. A 100% response rate was achieved towards our study. The information collected by interviewed questionnaire was transferred to IBM SPSS 20.0 statistical software for analyzing the significance of the obtained results. The categorical variables including obtainability of healthcare facilities, compliance towards the treatment, limitations of the commute from distant areas, and reservations to opt a male healthcare provider were recorded.

The female research assistant was ensured, keeping in mind the cultural norms. Keeping in mind the ethical considerations regarding rules and regulations of the hospital, three principles were held – an institutional review committee clearance was obtained by Gulab Devi Chest Hospital authority, a written consent form was signed by the correspondents of the interview and a non-coercive approach was adopted by the interviewers. Each study participant was assured of the confidentiality of their answers and selective individuals had access to the patient information.

## Results

In our study, we compared the association of level of education and age with stigma, reluctance to face male care providers, and requiring permission for going to the health facility.

Among the total participants of the study, 61% (169 out of 277) were illiterate, while 12% (32 out of 277) were literate. Eight percent (22 out of 277) had less than primary level education, 16% (45 out of 277) had less than matric level education, two percent (five out of 277) had education up to graduation, and only one percent (three out of 277) did not answer. Furthermore, 91% (251 out of 277) of the participants had their residence at a distance of more than 30 minutes drive, which showed the positive attitude of participants toward the health seeking behavior despite the long distance.This may also be due to the fact that there are very few specialized health care centers. Eight percent (22 out of 277) of the participants had their residence 15 to 30 minutes’ walk from the hospital. The results showed that most of the participants (91%; 253 out of 277) have to hire a transport to reach hospital which again showed a positive attitude keeping in view that all participants belong to a very low socioeconomic class. Five percent (13 out of 277) owed their own transport, four percent (10 out of 277) traveled by foot. One person gave no response (one out of 277).

Sixty-five percent (180 out of 277) required permission from family for going to the health facility and 35% (97 out of 277) never required permission. In the category of people who required permission, 58% (105 out of 180) required permission from their husbands, Seven percent (13 out of 180) required permission from their in-laws, 34% (61 out of 180) required permission from their parents. One participant (one percent; one out of 180) didn’t choose to answer the question.

In this study from a total of 277 individuals studied, 48% (134 out of 277) felt insecure in traveling alone to the health facility, while 47% (130 out of 277) felt secure. A number of participants (five percent; 13 out of 277) gave no response. There was a mixed response of the participants about feeling stigmatized when telling someone about their disease. From the total of 277 participants, 40% (112 out of 277) said that they were stigmatized and 54.5% (151 out of 277) said that they did not feel stigmatized when they had to tell someone about their disease. 5.1% (14 out of 277) didn’t answer.

In response to their reluctance towards a male care provider, a popular societal idea was challenged and a positive shift was seen. The majority of the females, (65%; 180 out of 277) said they did not feel reluctant facing a male health care provider, 30% (83 out of 277) said that they have certain reservations. Five percent (14 out of 277) refused to answer this question. Comparing education with stigma, the study revealed that 42% (45 out of 108) of the literate women felt stigmatized while 39% (65 out of 169) of illiterate women felt stigmatized. A test of significance was applied, chi-square, which showed the value of 0.275 and p-value of 0.600, which proved to be of less significance.

Comparing education with requiring permission for going to health facility, the study revealed that 62% (67 out of 108) of the literate women required permission while 67% (113 out of 169) of the illiterate women required permission. Although educated, women were still bound by the norms of the society and required permission to go to the health facility. Chi-square value of 0.675 (p-value of 0.411), indicating no significant difference between illiterate and literate women in seeking permission from home to visit a health facility was calculated.

## Discussion

Poorest of people from the poorest of countries are the ones mostly affected by TB. Not only are they more vulnerable to the disease because of their living and working conditions, they also seem to be plunged deeper into poverty as a consequence of TB [8]. Barriers to early detection and treatment of TB may be greater for women than for men [9].

According to a study carried in Nishtar Medical College Hospital, Multan, Pakistan, it was concluded that female to male ratio was 1.05:1 for tuberculosis in an out-patient clinic. This was diagnosed based on sputum examination. Although this ratio was recorded to be less based on acid-fast bacilli smear (females to male ratio, 0.84:1) [4].

This study was exclusively conducted to assess the health-seeking behavior of women affected with TB. We studied a number of factors which could influence the health-seeking behavior of females including educational status, distance of health facility from the house, requiring permission for going to the health facility for the treatment, feeling insecurity in going to the health facility, inability to go to health facility due to daily workload, reluctance to face male care providers, and feeling stigmatized about their disease. A study conducted in Delhi showed that there was no significant difference in health seeking behavior in relation to literacy status [10].

Moreover, amongst the participants studied, the majority of them (251; 90.6%) had their residence at a distance of more than 30-minute walk, which showed the positive attitude of participants toward the health seeking behavior despite the long distance. Furthermore, of the 277 individuals studied, 180 (65%) required permission for going to the health facility and among them, 105 (58.3%) required permission from their husbands, 13 (7.2%) from their in-laws and 61 (33.8%) from their parents. On the other hand, a study conducted in Sindh province in July 2003 showed that 53% of the rural and 33.6% of the urban women needed their husbands to go to a health facility along with them [11]. Pakistan and most of the other developing countries are male dominant societies where females do not have equal rights as males and this gender bias is a barrier in the healthcare seeking behavior of females [5]. In most households, it is mandatory that the females are accompanied by males to go to any health facility [12-13].

There was a mixed response of the participants about feeling stigmatized when telling someone about their disease. From the total of 277 participants, 41% said that they were stigmatized and 54.5% said that they did not feel stigma when they told someone about their disease, although different studies showed that the stigma associated with the disease is prevalent in different parts of the world [14].

### Limitations

It is considered that the study was non-randomized and cross-sectional, that may be less relatable in the measure of its credibility among masses. Furthermore, statistical relevance could have established evidence-based details from the data. Another important thing that the study could not address is the ways of minimizing Hawthorne effect. In general, the study does provide many informative details that can guide public health leaders.

## Conclusions

One major finding from the study was that females were keen to present their symptoms to the care providers despite long distances from the health units. It was also found that the majority of the females were bound to seek permission from the senior family members before visiting these health facilities. This permission seeking behavior was common in the majority of the study participants, irrespective of their education status. It was incredible to see a social shift, where the females motioned to be treated by male doctors without any hesitation.
